# Erythromelalgia and Its Allies

**Published:** 1905-04-01

**Authors:** 


### ERYTHROMELALGIA AND ITS ALLIES.

Dr. Vilches[1] has recently reviewed the vasomotor neuroses which attack the extremities. These are represented on the paralytic side by the condition to which Weir-Mitchell gave the name Erythromelalgia, and on the spasmodic side by the varying stages of peripheral anæmia which are generally embraced by the term Raynaud's disease. Dr. Vilches has attempted to show that the true counterpart of erythromelalgia is not Raynaud's disease, but a state of minor arterial spasm for which he proposes the name leucomelalgia; but we may dismiss the distinction, for it appears to be founded on the assumption that peripheral anæmia is not Raynaud's disease until a condition of gangrene is established. It seems quite clear that the matter at issue is only a question of degree.

Erythromelalgia had been several times described in the literature before its christening by Weir-Mitchell; amongst others by Vulpian in 1875, who set out all its characteristic features in detail. It consists, as its name implies, of a painful redness of one extremity or more, it affects males rather than females, and the lower extremity rather than the upper; elevation of the affected limb reduces the redness and relieves the pain, but as soon as a dependent position is resumed the skin becomes a brilliant red, and ultimately a deep purple. The digits alone may suffer, or the area of involvement may include larger districts, and even the whole limb. Occasionally, as in a case mentioned by Barlow,[2] a limited area in any part of the foot may be alone affected. The attack comes on more or less suddenly, and may last a few minutes or many hours. In a bad case recumbency may relieve the pain but little, and the hyperæsthesia of the skin may render the weight of the bedclothes intolerable. Occasionally the disease assumes a cyclical character, the attacks progressing in severity to a climax which is followed by a slow abatement, and a period of immunity. Although the ailment is exceedingly distressing, the prognosis is good and leaves no residual lesions as a rule.

The reverse—namely, the spasmodic condition, is much more debatable ground. The lesion originally described by Raynaud was a symmetrical gangrene of the extremeties believed to be due to arterial spasm and chiefly affecting the young. In order to establish the diagnosis of Raynaud's disease, as now generally understood (*i.e.* a pure vaso-constrictor anæmia of the extremities unattended by permanent alteration in the arterial trunks), it is necessary to eliminate three other conditions at least. These are, firstly, that of which senile gangrene is the type, that is, an arterial blocking due to atheroma. Secondly, diabetic gangrene. Thirdly, the gangrene of the extremities in the young, commonly called the arteritis obliterans of Friedländer (though, it may be said, the lesion is not that which Friedländer implied by his term). The distinction, however, does not as a rule present much difficulty. Atheroma is an uncommon lesion at the time of life at which Raynaud's disease is likely to be met with. Also, its evidences are not identical with those of the latter disease, and palpable evidence of the arterial sclerosis is almost certain to be available. Diabetic gangrene has only to be remembered to be identified. The third condition, to define which v. Winiwarter perverted Friedländer's "arteritis obliterans," is asymmetrical, not spasmodic but progressive, and the pulselessness or diminished pulsation of the arterial trunks to the affected extremity are constant features.

The characteristics of the true Raynaud's disease are the following. A spasmodic vaso-constriction of the extremities, chiefly the upper extremities, leading to "deadness" of the digits and parts affected, the areas involved being, as a rule, accurately symmetrical, and either dead-white, cyanotic, or gangrenous according to the severity and duration of the spasm. It is by election a disease of the first half of adult life, and of females far more often than of males. The arteries affected, though narrowed in volume during the attack, return to the normal in the intervals, and show no sign of tissue-change. The attacks of spasm are particularly prone to be determined by exposure to cold, and are therefore more frequent during the winter months. Lastly, the attacks are often associated with paroxysmal hæmoglobinuria. Dickinson[3] considers them both to be the expression of the same condition, and has seen them alternate in the same patient.

As to treatment, Dr. Vilches speaks very highly of galvanism. He says that currents of moderate strength contract blood-vessels, while stronger ones dilate them; also that contraction is marked in the cathodal region, dilatation in the anodal. The mode of application recommended by him is as follows:—In the case of erythromelalgia, the sympathetic in the neck is strongly galvanised for three minutes; this is followed by galvanism of the brachial plexus for five minutes, the anode being on the nape of the neck. Finally, the whole extremity is submitted to the action of the cathode. In this way he claims to have cured one case in less than three weeks, and to have greatly improved another in fifteen days. The treatment he advocates for the spasmodic conditions is practically the same, except that the anode is in this case applied to the structures above mentioned and that the final galvanism of the extremities is replaced by energetic faradisation. Barlow also advises galvanism for Raynaud's disease, but recommends its application through water in an arm-bath. Chemical vaso-dilators are of little service.

[1] Revista Ibero-American de Ciencias Med., Sept., 1904. [2] Allbutt's System of Med., VI.: "Erythro-melalgia." [3] Renal and Urinary Affections, Part III., 1885.

